# Dietary inflammatory index (DII) may be associated with hypertriglyceridemia waist circumference phenotype in overweight and obese Iranian women: a cross sectional study

**DOI:** 10.1186/s13104-021-05712-7

**Published:** 2021-08-16

**Authors:** Atefeh Tavakoli, Atieh Mirzababaei, Hanieh Moosavi, Sanaz Mehranfar, Seyed-Ali Keshavarz, Khadijeh Mirzaei

**Affiliations:** 1 Department of Community Nutrition, School of Nutritional Sciences and Dietetics, Tehran University of Medical Sciences (TUMS), P.O. Box: 14155-6117, Tehran, Iran; 2 Department of Clinical Nutrition, School of Nutritional Sciences and Dietetics, Tehran University of Medical Sciences, Tehran, Iran

**Keywords:** Dietary inflammatory index, Hypertriglyceridemic waist circumference phenotype, Obesity

## Abstract

**Objective:**

Recent studies have shown that increased dietary inflammatory index (DII) score or consumption of pro-inflammatory foods can lead to increased waist circumference (WC) as well as triglyceride (TG) concentrations in obese people. The purpose of this study is to examine the association between DII and hypertriglyceridemic waist circumference phenotype (HTGWCP) in women with overweight and obesity.

**Results:**

There was a positive significant correlation between DII and HTGWCPs. In other words, with an increase in DII score or higher consumption of pro-inflammatory foods, the odds of having abnormal phenotypes including; enlarged waist normal TG (EWNT) (OR = 2.85, 95% CI 1.02 to 7.98, P for trend = 0.04), normal waist enlarged TG (NWET) (OR = 5.85, 95% CI 1.1 to 31.11, P for trend = 0.03) and enlarged waist enlarged TG (EWET) (OR = 3.13, 95% CI 0.95 to 10.27, P for trend = 0.05) increase compared to normal waist normal TG (NWNT) phenotype. In conclusion; increasing DII scores can increase abnormal phenotypes and therefore may increase WC and TG levels in overweight and obese women.

**Supplementary Information:**

The online version contains supplementary material available at 10.1186/s13104-021-05712-7.

## Introduction

Obesity is an important public health issue in all over the world and it is emerging as a threat in more affluent sectors of developing countries [1]. According to the World Health Organization (WHO) in 2008, more than10% of the world's adult population, about 500 million people were obese [2]. This issue is associated with chronic diseases including; cardiovascular disease (CVD), diabetes, breast and endometrial cancer in women [1]. Obesity is usually caused by the accumulation of excess fat over the body and it is often characterized as a state of low-grade chronic inflammation [3].

Many studies have shown an association between diet and inflammatory markers and chronic inflammatory-related diseases such as obesity [4–6]. Healthy dietary patterns, such as the Mediterranean diet (MeDiet), can reduce the low-grade inflammation observed in obese persons due to some of their components are known as an anti-inflammatory dietary pattern [7–10]. The DII as a new tool to assess inflammatory potential of the diet determines the inflammatory potential of the diet on a continuum from maximally anti- to pro-inflammatory [11]. The results of a study showed that DII was directly associated with the incidence of obesity, especially abdominal obesity in women compared to men with higher body mass index (BMI), WC, waist-to-height ratio (WHtR). According to results of this study, DII was inversely related with the consumption of anti-inflammatory nutrients and foods [12, 13]. A study concluded that unhealthy diets such as the Western diet, which are associated with higher inflammation, increased metabolic syndrome (MetS) components, such as WC and TG levels, compared with the MeDiet [4]. Several investigations resulted that the inflammatory properties of the diet were associated with higher TG and lower high-density lipoprotein (HDL) plasma levels [14, 15]. The HTGWCP is defined as high serum TG levels and simultaneous presence of high WC and also it is a marker for assessing visceral obesity and low-grade inflammation status such as obesity [16]. Due to the ascending growth of obesity and the strong association of this chronic inflammatory condition with many other chronic diseases [1], as well as the results of many previous studies, especially cohort studies [15, 17], which indicate the relationship between pro-inflammatory diet patterns and metabolic syndrome components, especially WC, HDL and TG, as well as some other anthropometric indices such as fat free mass (FFM), we weighed to examine the association of DII with obesity [18, 19]. On the other hand, studies have shown that following pro-inflammatory diets raises the levels of many inflammatory markers, including; Tumor necrosis factor alpha (TNFα), high-sensitivity C-reactive protein (hs-CRP), Interleukin1beta (IL-1β), Interleukin 6 (IL-6), Interleukin 10 (IL-10), in obese or chronically ill people, especially in women, which in turn has led to the spread of obesity in these people [13, 20, 21]. Due to the prevalence of obesity, abdominal obesity and malnutrition in developing societies, especially among women, as well as the limitations of studies conducted on this sensitive group of society [22–24] and also the greater likelihood of the spread of inflammatory conditions, such as viral [25] and respiratory inflammatory diseases [26] in women compared to men and also according to our investigations, the majority of studies on the relationship between DII and obesity and many other diseases are more in women compared to men [27–29]. In this regard, a 20-year cohort study was performed on 70,991 women with diabetes and the effect BMI mediator was also examined in this study, the results of which showed a direct relationship between the incidence of diabetes and DII through BMI [30]. So, we selected women as the target group.

There is no investigation on the association between DII and HTGWCP, we aimed to evaluate the relationship between DII and HTGWCP among obese and overweight women.

## Main text

### Method

#### Study population

226 women aged 18 to 48 years were participated. The sample size was calculated based on the following formula: n = [(Z 1 − α + Z 1 − β) × √1 − r2]/r) 2 + 2), which α = 0.05, β = 0.95, r = 0.25, and then, with 80% power and 95% confidence. BMI range for women participants was between 25 to 49.6 kg/m^2^. Inclusion criteria: aged 18 to 48 years, overweight or obese women (BMI ≥ 25 kg/m^2^) and exclusion criteria: menopausal pregnancy or lactation period, diabetes mellitus, liver and kidney disorders, hypertension, CVD, smoking and alcohol consumption, chronic diseases affecting the dietary pattern. Participating women signed the consent form. The protocol was accepted by the Ethical Commission at Tehran University of Medical Sciences (IR.TUMS.MEDICINE.REC.1399.636).

#### Anthropometric assessment

Weight was measured with the use of a digital scale (Seca, Hamburg, Germany) with the least clothes and without shoes. Height was measured using a calibrated height gauge (in the standing position without shoes). hip circumference (HC) and WC were measured in the largest and the smallest girth, respectively. BMI was calculated as weight (kg) to height (m^2^) ratio. skeletal muscle mass (SMM), FFM, waist hip ratio (WHR) as body composition components; evaluated using a body composition analyzer (InBody770 scanner; InBody, Seoul, Korea).

#### Biochemical assessment

After 10 to 12 h of fasting, blood samples were taken and the serum was stored at − 80 °C. All samples were analyzed by using a single assay according to manufacturer’s protocol. Glucose oxidase method was using for fasting plasma glucose assessment and insulin level was measured by using an enzyme-linked immunosorbent assay (ELISA) kit (Human insulin ELISA kit, DRG Pharmaceuticals, GmbH, Germany). TG, Total cholesterol (T-Chole), Low-density lipoprotein (LDL), HDL were measured by related kits (Pars Azemun, Iran). serum glutamic-pyruvic transaminase (SGPT) and serum glutamic-oxaloacetic transaminase (SGOT) were assessed using the International Federation of Clinical Chemistry and Laboratory Medicine standardization. The Homeostatic model assessment for insulin resistance (HOMA-IR) was estimated as the product of fasting glucose and insulin level divided by 22.5 with molar unit (mmol/L).

#### Demographic variables

Demographic questionnaire was used for evaluation of qualitative variables such as education, job, marital situation, economic status and familial obesity history.

#### Dietary inflammatory index

More negative DII or a lower DII score indicates eat less inflammatory foods and more positive DII or a higher DII score indicates eat more inflammatory foods. Dietary intake was assessed by using a semi-quantitative food frequency questionnaire (FFQ) contains 147 Iranian food points, with a list of foods and their standard serving sizes that assesses the usual food intake in the past year. The validity and reliability of FFQ have been confirmed [31]. Software program Nutritionist IV was used for nutrient analysis which was modified for Iranian foods. DII was computed based on Shivappa et al. and Esfahani et al. methods [32, 33]. To calculate DII, first, the Z-score was computed by subtracting the global standard mean of the declared food amounts and so dividing the difference by the global standard deviation. After that, the Z-score was converted to a centered percentile score and this was multiplied by the food parameter effect score, to obtain a DII score for a subject. To create the final DII score, all of the food parameter-specific DII scores were summed.

#### Hypertriglyceridemic waist circumference phenotype

Women were categorized in 4 phenotype groups according to the cutoff points: NWNT: normal WC < 88 cm and normal serum TG concentrations < 150 mg/dL), EWNT: enlarged WC ≥ 88 cm and normal serum TG concentration < 150 mg/dL), NWET: normal WC < 88 cm and elevated TG ≥ 150 mg/dL, EWET: enlarged WC ≥ 88 cm and hyper serum TG concentration ≥ 150 mg/dL [34].

#### Statistical analyzes

The normal distribution of data was checked by Kolmogorov Smirnov test. Quantitative variables were described using mean and standard deviation and qualitative variables by reporting number and percentage. Analysis of variance (ANOVA) test was confirmed for calculating of relationships between DII and HTGWCP and quantitative variables and also the tukey’s post hoc test was using for the adjustment of multiple comparisons. Relationships of DII, HTGWCP and qualitative variables was conducted by Chi-square test. Analysis of covariance (ANCOVA) was used to modify the effect of confounders included: age, energy intake, BMI and physical activity. Associations between DII and HTGWCP were confirmed in Multinomial logistic regression to adjust for confounder points; energy intake, age, insulin plasma level, marital status, education, economic status and family history of obesity. We considered the group of “NWNT” about HTGWCPs as reference. Statistical analyzes were performed using IBM SPSS version 25.0 (SPSS, Chicago, IL, USA). The level of significance was considered as being a P value ≤ 0.05.

### Result

#### Study population characteristics

The mean ± standard deviation (SD) of age, weight, BMI, WC were 36.67 ± 9.10 years, 81.29 ± 12.43 kg, 31.26 ± 4.29 kg/m^2^, 99.61 ± 10.07 cm, respectively. NWNT, EWNT, NWET, EWET phenotypes included 6.2%, 42%, 5.9, 45% of individuals respectively. 70.8% of women were married, 84.7% of them had a university education and 61.9% of them were unemployed.

#### Correlation analysis

After controlling the confounding variables including age, energy intake, BMI and physical activity: a significant correlation was found between DII and TG, and this variable had a higher mean in the second tertile of DII (P = 0.02) Table 1, Additional file 1: Table S1.

**Table 1:** Description of characteristics among tertiles of DII

Variables	T1 N = 76		T2 N = 77	T3 N = 73	P value^*^		P value^**^
Age (year)	37.03 (8.52)^a^		37.06 (8.22)	34.82 (8.43)	0.12		0.08
PA (MET h/week)	1200.87 (1376.69)		1173.54 (1555.11)	1143.56 (2725.58)	0.98		0.97
Blood parameters							
FBS (mg/dL)		85.74 (8.06)	88.51 (10.31)	87.22 (10.50)	0.21	0.13	
Insulin (µIU/mL)		1.26 (0.23)	1.24 (0.21)	1.22 (0.25)	0.63	0.99	
HOMA-IR		3.63 (1.62)^3^	3.19 (1.25)	2.87 (0.93)^1^	**0.02**^**a**^	0.09	
TC (mg/dL)		184.02 (39.14)	188.66 (33.93)	182.42 (35.14)	0.54	0.42	
HDL-C (mg/dL)		47.37 (13.06)	46.01 (10.10)	46.77 (10.38)	0.75	0.74	
LDL-C (mg/dL)		95.12 (26.36)	97.12 (23.05)	91.69 (21.95)	0.36	0.25	
TG (mg/dL)		145.91 (68.73)	108.09 (43.03)	108.84 (61.55)^1,2^	**0.002**^**b**^	**0.01**	
SGOT (mg/dL)		17.00 (5.99)	18.54 (9.19)	17.93 (6.68)	0.44	0.42	
SGPT (mg/dL)		17.60 (9.72)	20.20 (16.12)	19.42 (11.63)	0.44	0.49	
Body composition parameters							
BMI (kg/m^2^)		31.09 (3.88)	30.37 (4.65)	31.25 (4.10)	0.33	0.11	
SMM (kg)		25.67 (2.98)	25.54 (3.29)	25.66 (3.82)	0.96	0.95	
FFM (kg)		46.74 (5.02)	46.60 (5.58)	46.72 (6.49)	0.98	0.94	
WHR		1.94 (9.59)	0.93 (0.05)	0.93 (0.05)	0.37	0.85	
WC (cm)		98.97 (9.45)	97.76 (10.19)	99.52 (10.18)	0.48	0.38	
Qualitative variables							
Economic status							
Poor		12 (41.4%)^b^	7 (20.7%)	11 (37.9%)	0.67^***^	0.77	
Moderate		35 (34.8%)	33 (33.9%)	32 (31.3%)			
Good		29 (32.8%)	32 (34.4%)	29 (32.8%)			
Excellent		2 (12.5%)	5 (50%)	3 (37.5%)			
Education status							
Illiterate		1 (33.3%)	1 (33.3%)	1 (33.3%)	0.46	0.37	
Diploma		11 (38.9%)	7 (19.4%)	12 (41.7%)			
University educated		64 (33%)	69 (35.3%)	60 (31.7%)			
Marriage status							
Single		14 (26.8%)	20 (39.3%)	19 (33.9%)	0.38	0.17	
Married		62 (35.7%)	57 (31.4%)	63 (32.9%)			
Familial obesity history							
Yes		55 (34.2%)	56 (34.7%)	50 (31/1%)	0.24	0.16	
No		21 (31.1%)	18 (27.3%)	23 (41.6%)			
Hypertriglyceridemic waist circumference phenotype							
NWNT		3 (16.7%)	5 (27.8%)	8 (55.5%)	0.26	0.19	
EWNT		21 (29.2%)	32 (39.3%)	25 (31.5%)			
NWET		7 (30.4%)	8 (34.8%)	8 (34.8%)			
EWET		45 (38.6%)	32 (30.0%)	32 (31.4%)			

Bold values indicate the significant range as a tabular foot

T1, T2 and T3 are DII tertiles

*DII* dietary inflammatory index, *PA* physical activity, *FBS* Fasting blood sugar, *HOMA-IR* Homeostatic Model Assessment for Insulin Resistance, *TC* total cholesterol, *HDL-C* high density lipoprotein cholesterol, *LDL-C* low density lipoprotein cholesterol, *TG* triglyceride, *SGOT* serum glutamic-oxaloacetic transaminase, *SGPT* Serum glutamic-pyruvic transaminase, *BMI*: body mass index, *SMM* Skeletal muscle mass, *FFM* fat free mass, *WHR* waist to hip ratio, *WC* waist circumference, *NWNT* normal waist normal triglyceride, *EWNT* enlarged waist normal triglyceride, *NWET* normal waist enlarged triglyceride, *EWET* enlarged waist enlarged triglyceride

* P value resulted from ANOVA analysis

** P value reported after adjusting age, energy intake, BMI and physical activity with ANCOVA

*** P value resulted from chi-squared test analysis

^a^Mean ± SD

^b^N(%), P value ≤ 0.05 is significant

After adjustment for confounders including; age, energy intake, BMI and physical activity through ANCOVA test insulin (P = 0.01) and HOMA-IR, TG, weight, height, BMI, FFM, WHR, WC (P < 0.001) and SMM (P = 0.002), were all significant with HTGWPs and TG concentrations, weight, BMI, SMM and FFM were higher in EWET phenotype, insulin and HOMA-IR were higher in NWET phenotype and height, WHR and WC were higher in EWNT phenotype Table 2, Additional file 2: Table S2.

**Table 2:** Description of characteristics among types of hypertrigeliceridemic waste phenotype

Variables	Hypertriglyceridemic waist circumference phenotypes					P value^*^	P value^**^
	NWNT N = 15		EWNT N = 88	NWET N = 14	EWET N = 109		
Age (year)	32.08 ± 8.08^a,4^		36.87 ± 9.78	36.20 ± 7.18	37.17 ± 8.66^1^	0.06	0.17
PA (MET h/week)	682 ± 832.81		973.86 ± 1158.23	1972 ± 4117.85	1277.48 ± 2171	0.29	0.22
Blood parameters							
Insulin (µIU/mL)	1.21 ± 0.18		1.19 ± 0.23^4^	1.37 ± 0.18	1.29 ± 0.24^2^	**0.01**	**0.01**
HOMA-IR	3.32 ± 1.35^4^		3.09 ± 1.03^3,4^	4.72 ± 1.32^2^	4.14 ± 1.56^1,2^	** < 0.001**	** < 0.001**
TC (mg/dL)	168.81 ± 26.56		188.05 ± 38.15	173.37 ± 29.49	187.70 ± 35.52	0.06	0.73
HDL-C (mg/dL)	49.56 ± 10.71		45.75 ± 11.16	46.66 ± 11.51	46.74 ± 10.60	0.63	0.97
LDL-C (mg/dL)	86.68 ± 18.82		94.70 ± 26.51	93.41 ± 20.33	97.10 ± 23.65	0.40	0.76
TG (mg/dL)	93.32 ± 27.13^3,4^		92.34 ± 27.79^3,4^	189.00 ± 30.41^1,2^	213.52 ± 49.52^1,2^	** < 0.001**	** < 0.001**
SGOT (mg/dL)	17.62 ± 5.48		18.11 ± 7.72	16.29 ± 5.05	18.40 ± 8.41	0.67	0.63
SGPT (mg/dL)	13.93 ± 6.24		20.22 ± 13.92	17.37 ± 8.01	20.09 ± 15.13	0.30	0.62
Body composition parameters							
Weight(cm)	65.88 ± 3.45^2,4^		83.06 ± 11.68^1,3^	65.44 ± 3.01^2,4^	83.85 ± 11.64^1,3^	** < 0.001**	** < 0.001**
Height(cm)	159.26 ± 4.84		161.78 ± 5.79^3^	156.70 ± 4.79^2,4^	161.55 ± 5.96^3^	** < 0.001**	** < 0.001**
BMI (kg/m^2^)	25.96 ± 0.86^2,4^		31.76 ± 4.13^1,3^	26.72 ± 1.26^2,4^	32.11 ± 4.12^1,3^	** < 0.001**	** < 0.001**
SMM (kg)	22.08 ± 2.19^2,4^		25.75 ± 3.14^1,3^	22.48 ± 1.95^2,4^	26.26 ± 3.52^1,3^	** < 0.001**	**0.002**
FFM (kg)	40.81 ± 3.66^2,4^		46.79 ± 5.09^1,3^	41.44 ± 3.39^2,4^	47.73 ± 5.93^1,3^	** < 0.001**	** < 0.001**
WHR	0.86 ± 0.18		1.48 ± 6.98	0.86 ± 0.02	0.94 ± 0.04	0.68	** < 0.001**
WC (cm)	84.66 ± 2.16^2,3,4^		101.74 ± 8.99^1,3^	85.02 ± 1.85^2,4^	101.60 ± 8.97^1,3^	** < 0.001**	** < 0.001**
Qualitative variables							
Economic status							
Poor	2 (5.0%)^b^		13 (32.5%)	2 (5.0%)	20 (57.5%)	0.18^***^	0.65
Moderate	7 (7.8%)		33 (41.9%)	6 (6.6%)	47 (43.7%)		
Good	5 (5.8%)		27 (41.3%)	6 (7.1%)	38 (45.8%)		
Excellent	1 (5.0%)		15 (75.1%)	0 (0%)	4 (19.9%)		
Education status							
Illiterate	0 (0.0%)		1 (25.0%)	1 (25.0%)	4 (50.0%)	0.31	0.88
Diploma	1 (2.0%)		17 (36.7%)	2 (4.1%)	31 (57.2%)		
University educated	14 (7.0%)		70 (43.3%)	11 (6.1%)	74 (43.6%)		
Marital status							
Single	5 (7.3%)		36 (52.3%)	3 (3.7%)	29 (36.7%)	**0.05**	0.47
Married	10 (5.9%)		52 (38.5%)	11 (7.0%)	80 (48.6%)		
Familial obesity history							
Yes	7 (5.2%)		56 (39.7%)	9 (6.7%)	85 (48.4%)	0.17	0.92
No	8 (9.3%)		32 (47.2%)	5 (5.5%)	24 (38%)		

Bold values indicate the significant range as a tabular foot

*NWNT* normal waist normal triglyceride, *EWNT* enlarged waist normal triglyceride, *NWET* normal waist enlarged triglyceride, *EWET* enlarged waist enlarged triglyceride, *PA* physical activity, *HOMA-IR* Homeostatic Model Assessment for Insulin Resistance, *TC* total cholesterol, *HDL-C* high density lipoprotein cholesterol, *LDL-C* low density lipoprotein cholesterol, *TG* triglyceride,* SGOT* serum glutamic-oxaloacetic transaminase, *SGPT* Serum glutamic-pyruvic transaminase, *BMI* body mass index, *SMM* skeletal muscle mass, *FFM* fat free mass, *WHR* waist to hip ratio, *WC* waist circumference

* P value resulted from ANOVA analysis

** P value reported after adjusting age, energy intake, BMI and physical activity with ANCOVA analysis

*** P value resulted from chi-squared test analysis

^a^Mean ± SD

^b^N(%), P value ≤ 0.05 is significant

In crude model a significant correlation was showed between DII and EWET (OR = 2.07, 95% CI 0.42 to 0.69, P for trend = 0.02). By adjusting variables included; energy intake, age, plasma insulin levels, marital status, education, economic status and familial obesity history, we observed that with increasing DII, the odds of having the EWNT (OR = 2.85, 95% CI 0.77 to 0.86, P for trend = 0.04), NWET (OR = 5.85, 95% CI 0.56 to 0.72, P for trend = 0.03) and EWET (OR = 3.13, 95% CI 0.86 to 0.98, P for trend = 0.05) phenotypes were significantly increased compared to NWNT phenotype among participants Table 3, Additional file 3: Table S3.

**Table 3:** Crude model and adjusted model for Relationship between hyper triglyceridemic waist circumference phenotype and DII

	OR (95% CI)	β ± SE	P-trend
Crude model			
EWNT	1.80 (0.91 to1.01)	0.58 ± 0.34	0.08
NWET	1.74 (0.78 to 2.09)	0.55 ± 0.4	0.17
EWET	2.07 (0.42 to 0.69)	0.73 ± 0.33	**0.02**
Adjusted model^a^			
EWNT	2.85 (0.77 to 0.86)	1.04 ± 0.52	**0.04**
NWET	5.85 (0.56 to 0.72)	1.76 ± 0.85	**0.03**
EWET	3.13 (0.86 to 0.98)	1.14 ± 0.6	**0.05**

Bold values indicate the significant range as a tabular foot

Normal waist normal triglyceride (NWNT) Consider as reference

*DII* dietary inflammatory index, *OR* odds ratio, *CI* confidence interval, *SE* standard error, *EWNT* enlarged waist normal triglyceride, *NWET* normal waist enlarged triglyceride, *EWET* enlarged waist enlarged

^a^Adjusted for energy intake, age, plasma insulin levels, marriage status, educational status, economic status and familial obesity history, P value ≤ 0.05 is significant

### Discussion

The present investigation was the first cross-sectional study that evaluated the association between DII and HTGWCP among overweight and obese women. We have shown that DII is significantly associated with HTGWCP. Our findings suggested that, increasing DII scores or consuming pro-inflammatory foods can increase the odds of abnormal phenotypes including; EWNT, NWET, EWET phenotypes compared to NWNT phenotype. According to the results of this study; there was a significant correlation between DII and TG (negatively) so in this study increased DII did not increase TG. However, there was a positive significant relationship between abnormal phenotypes and variables such as insulin, HOMA-IR, TG, weight, height, BMI, SMM, FFM, WHR and WC.

Previous studies have shown that obesity incidence and high WC can be a consequence of previous chronic low-grade inflammation. Therefore, there is a relationship between obesity, especially abdominal obesity and inflammation [35]. Some studies acknowledge that abnormal lipid metabolism plays an important role in the inflammatory response and prognosis of the conditions like obesity and hypertriglyceridemia [19, 36]. In a cohort study, it was found that the HTGWCP had a prevalence similar to MetS among participants and was associated with the low-grade inflammation seen in obese individuals [37]. Many studies have been shown that DII was correlated with anthropometric components such as weight and WC, and by increasing this score, these components have also increased. A study has shown that the pro-inflammatory diet had a stronger association with WC than with other anthropometric components, among women and men [17, 18]. Many studies in Western societies suggest that the inflammatory properties of diet are positively associated with predictors of CVD, such as increased plasma TG levels and decreased HDL concentrations. Neufcourt et al. showed in a large (n = 3726) cohort of French adults that after a follow up of 13 years, the DII score was significantly associated with higher TG and lower HDL levels [14, 15, 38]. One investigation concluded that among police officers, higher DII scores were associated with elevated hs-CRP values and the components of MetS [39].

Our study may be considered as an effective way to prevent the development of obesity. We also evaluated the relationship between some body composition indicators and biochemical parameters and DII, as well as HTGWCP. We selected our target group as women as one of the most sensitive groups in society. We tried to select and adjust some confounders socio-economically because of the association between hypertriglyceridemia and abdominal obesity and such factors.

### Conclusion

It was shown that an increase in DII scores or higher consumption of pro-inflammatory foods can increase all three abnormal phenotypes (EWNT, NWET, EWET) so it may lead to an increase in WC as an important indicator in obesity studies and TG concentration among overweight and obese women. Based on the study, it is recommended that this relationship be done in other target groups such as male adolescent children, etc. It is also better to find more causal clinical studies in this field to find a causal relationship. In addition, researchers can communicate examine many dietary patterns with these phenotypes in their studies.

## Limitation

Our study also had its limitations. Briefly, since the study was a cross-sectional study, causal relationship was not investigated, only correlation. We calculated the DII using FFQ that is recognized to have over-reporting and under-reporting bias.

## Supplementary Information

**Additional file 1: Table S1.** Description of characteristics among tertiles of DII.

**Additional file 2: Table S2.** Description of characteristics among types of hypertrigeliceridemic waste phenotype.

**Additional file 3: Table S3.** Crude model and adjusted model for Relationship between hyper triglyceridemic waist circumference phenotype and DII.

## Data Availability

The data that support the findings of this study are available from Dr. Khadijeh Mirzaei but restrictions apply to the availability of these data, which were used under license for the current study, and so are not publicly available. Data are however available from the authors upon reasonable request and with permission of Dr. Khadijeh Mirzaei.

## Abbreviations

DII: Dietary inflammatory index
WC: Waist circumference
HTGWCP: Hypertriglyceridemic waist circumference phenotype
TG: Triglyceride
EWNT: Enlarged waist normal TG
NWET: Normal waist enlarged TG
EWET: Enlarged waist enlarged TG
NWNT: Normal waist normal TG
WHO: World Health Organization
CVD: Cardiovascular disease
MeDiet: Mediterranean diet
BMI: Body mass index
WHtR: Waist-to-height ratio
MetSyn: Metabolic syndrome
HDL: High-density lipoprotein
FFM: Fat free mass
TNFα: Tumor necrosis factor alpha
hs-CRP: High-sensitivity C-reactive protein
IL-1β: Interleukin1beta
IL-6: Interleukin 6
IL-10: Interleukin 10
HC: Hip circumference
SMM: Skeletal muscle mass
WHR: Waist hip ratio
ELISA: Enzyme-linked immunosorbent assay
T-chole: Total cholesterol
LDL: Low-density lipoprotein
SGPT: Serum glutamic-pyruvic transaminase
SGOT: Serum glutamic-oxaloacetic transaminase
HOMA-IR: Homeostatic model assessment for insulin resistance
FFQ: Food frequency questionnaire
ANOVA: Analysis of variance
ANCOVA: Analysis of covariance
SD: Standard deviation
