# Supplementary material for: Dietary inflammatory index (DII) may be associated with hypertriglyceridemia waist circumference phenotype in overweight and obese Iranian women: a cross sectional study
Source: BMC Res Notes. 2021 Aug 16;14:312. doi: 10.1186/s13104-021-05712-7 (PMC8365886; doi:10.1186/s13104-021-05712-7)
Supplement: Supplementary file 1 — Additional file 1: Table S1. Description of characteristics among tertiles of DII. [file 13104_2021_5712_MOESM1_ESM.docx]

**Additional Material**

Additional file 1: Table S1: Description of characteristics among tertiles of DII.

| **Table S1.** **Description of characteristics among tertiles of DII** | | | | | | | |
| --- | --- | --- | --- | --- | --- | --- | --- |
| **Variables** | **T1**  **N=76** | | **T2**  **N=77** | **T3**  **N=73** | **P value^*^** | | **P value^**^** |
| Age (year) | 37.03(8.52)^a^ | | 37.06(8.22) | 34.82(8.43) | 0.12 | | 0.08 |
| PA (MET h/week) | 1200.87(1376.69) | | 1173.54(1555.11) | 1143.56(2725.58) | 0.98 | | 0.97 |
| **Blood parameters** | | | | | | | |
| FBS (mg/dL) | | 85.74(8.06) | 88.51(10.31) | 87.22(10.50) | 0.21 | 0.13 | |
| Insulin (µIU/ml) | | 1.26(0.23) | 1.24(0.21) | 1.22(0.25) | 0.63 | 0.99 | |
| HOMA-IR | | 3.63(1.62) ^3^ | 3.19(1.25) | 2.87(0.93)^1^ | **0.02^a^** | 0.09 | |
| TC (mg/dL) | | 184.02(39.14) | 188.66(33.93) | 182.42(35.14) | 0.54 | 0.42 | |
| HDL-C (mg/dL) | | 47.37(13.06) | 46.01(10.10) | 46.77(10.38) | 0.75 | 0.74 | |
| LDL-C (mg/dL) | | 95.12(26.36) | 97.12(23.05) | 91.69(21.95) | 0.36 | 0.25 | |
| TG (mg/dL) | | 145.91(68.73) | 108.09(43.03) | 108.84(61.55)^1,2^ | **0.002^b^** | **0.01** | |
| SGOT (mg/dL) | | 17.00(5.99) | 18.54(9.19) | 17.93(6.68) | 0.44 | 0.42 | |
| SGPT (mg/dL) | | 17.60(9.72) | 20.20(16.12) | 19.42(11.63) | 0.44 | 0.49 | |
| **Body composition parameters** | | | | | | | |
| BMI (kg/m^2^) | | 31.09(3.88) | 30.37(4.65) | 31.25(4.10) | 0.33 | 0.11 | |
| SMM (kg) | | 25.67(2.98) | 25.54(3.29) | 25.66(3.82) | 0.96 | 0.95 | |
| FFM (kg) | | 46.74(5.02) | 46.60(5.58) | 46.72(6.49) | 0.98 | 0.94 | |
| WHR | | 1.94(9.59) | 0.93(0.05) | 0.93(0.05) | 0.37 | 0.85 | |
| WC (cm) | | 98.97(9.45) | 97.76(10.19) | 99.52(10.18) | 0.48 | 0.38 | |
| **Qualitative variables** | | | | | | | |
| **Economic status** | | | | | | | |
| Poor | | 12(41.4%)^b^ | 7(20.7%) | 11(37.9%) | 0.67^***^ | 0.77 | |
| Moderate | | 35(34.8%) | 33(33.9%) | 32(31.3%) |  |  |  |
| Good | | 29(32.8%) | 32(34.4%) | 29(32.8%) |  |  |  |
| Excellent | | 2(12.5%) | 5(50%) | 3(37.5%) |  |  |  |
| **Education status** | | | | | | | |
| Illiterate | | 1(33.3%) | 1(33.3%) | 1(33.3%) | 0.46 | 0.37 | |
| Diploma | | 11(38.9%) | 7(19.4%) | 12(41.7%) |  |  |  |
| University educated | | 64(33%) | 69(35.3%) | 60(31.7%) |  |  |  |
| **Marriage status** | | | | | | | |
| Single | | 14(26.8%) | 20(39.3%) | 19(33.9%) | 0.38 | 0.17 | |
| Married | | 62(35.7%) | 57(31.4%) | 63(32.9%) |  |  |  |
| **familial obesity history** | | | | | | | |
| Yes | | 55 (34.2%) | 56 (34.7%) | 50 (31/1%) | 0.24 | 0.16 | |
| No | | 21 (31.1%) | 18 (27.3%) | 23 (41.6%) |  |  |  |
| **Hypertriglyceridemic waist circumference phenotype** | | | | | | | |
| NWNT | | 3(16.7%) | 5(27.8%) | 8(55.5%) | 0.26 | 0.19 | |
| EWNT | | 21(29.2%) | 32(39.3%) | 25(31.5%) |  |  |  |
| NWET | | 7(30.4%) | 8(34.8%) | 8(34.8%) |  |  |  |
| EWET | | 45(38.6%) | 32(30.0%) | 32(31.4%) |  |  |  |
| *^a^Mean±SD; ^b^ N(%); DII: dietary inflammatory index, PA: physical activity, FBS: Fasting blood sugar,*  *HOMA-IR:* *Homeostatic Model Assessment for Insulin Resistance, TC: total cholesterol, HDL-C: high density lipoprotein cholesterol, LDL-C: low density lipoprotein cholesterol, TG: triglyceride, SGOT: serum glutamic-oxaloacetic transaminase, SGPT: Serum glutamic-pyruvic transaminase, BMI: body mass index, SMM: Skeletal muscle mass, FFM: fat free mass, WHR: waist to hip ratio, WC: waist circumference, NWNT: normal waist normal triglyceride, EWNT: enlarged waist normal triglyceride, NWET: normal waist enlarged triglyceride, EWET: enlarged waist enlarged triglyceride*  **P value resulted from ANOVA analysis*  *** P value reported after adjusting age, energy intake, BMI and physical activity with ANCOVA*  **** P value resulted from chi-squared test analysis*  *T1, T2 and T3 are DII tertiles* | | | | | | | |
