# Supplementary material for: Dietary inflammatory index (DII) may be associated with hypertriglyceridemia waist circumference phenotype in overweight and obese Iranian women: a cross sectional study
Source: BMC Res Notes. 2021 Aug 16;14:312. doi: 10.1186/s13104-021-05712-7 (PMC8365886; doi:10.1186/s13104-021-05712-7)
Supplement: Supplementary file 2 — Additional file 2: Table S2. Description of characteristics among types of hypertrigeliceridemic waste phenotype. [file 13104_2021_5712_MOESM2_ESM.docx]

**Additional Material**

Additional file 2: Table S2: Description of characteristics among types of hypertrigeliceridemic waste phenotype.

| **Table S2: Description of characteristics among types of hypertrigeliceridemic waste phenotype** | | | | | | | | | | | | |
| --- | --- | --- | --- | --- | --- | --- | --- | --- | --- | --- | --- | --- |
| **Variables** | **Hypertriglyceridemic waist circumference phenotypes** | | | | | | | | **P value^*^** | | **P value^**^** | |
|  | **NWNT**  **N=15** | | | **EWNT**  **N=88** | **NWET**  **N=14** | **EWET**  **N=109** | | |  |  |  |  |
| Age(year) | 32.08 ± 8.08^a,4^ | | | 36.87 ± 9.78 | 36.20 ± 7.18 | 37.17 ± 8.66^1^ | | | 0.06 | | 0.17 | |
| PA (MET h/week) | 682±832.81 | | | 973.86±1158.23 | 1972±4117.85 | 1277.48±2171 | | | 0.29 | | 0.22 | |
| **Blood parameters** | | | | | | | | | | | | |
| Insulin (µIU/ml) | 1.21±0.18 | | | 1.19±0.23^4^ | 1.37±0.18 | | 1.29±0.24^2^ | | | **0.01** | | **0.01** |
| HOMA-IR | 3.32±1.35^4^ | | | 3.09±1.03^3,4^ | 4.72±1.32^2^ | | 4.14±1.56^1,2^ | | | **<0.001** | | **<0.001** |
| TC (mg/dL) | 168.81±26.56 | | | 188.05±38.15 | 173.37±29.49 | | 187.70±35.52 | | | 0.06 | | 0.73 |
| HDL-C (mg/dL) | 49.56±10.71 | | | 45.75±11.16 | 46.66±11.51 | | 46.74±10.60 | | | 0.63 | | 0.97 |
| LDL-C (mg/dL) | 86.68±18.82 | | | 94.70±26.51 | 93.41±20.33 | | 97.10±23.65 | | | 0.40 | | 0.76 |
| TG (mg/dL) | 93.32±27.13^3,4^ | | | 92.34±27.79^3,4^ | 189.00±30.41^1,2^ | | 213.52±49.52^1,2^ | | | **<0.001** | | **<0.001** |
| SGOT (mg/dL) | 17.62±5.48 | | | 18.11±7.72 | 16.29±5.05 | | 18.40±8.41 | | | 0.67 | | 0.63 |
| SGPT (mg/dL) | 13.93±6.24 | | | 20.22±13.92 | 17.37±8.01 | | 20.09±15.13 | | | 0.30 | | 0.62 |
| **Body composition parameters** | | | | | | | | | | | | |
| Weight(cm) | 65.88 ± 3.45^2,4^ | | 83.06 ± 11.68^1,3^ | | 65.44 ± 3.01^2,4^ | | 83.85 ± 11.64^1,3^ | | | **<0.001** | | **<0.001** |
| Height(cm) | 159.26 ± 4.84 | | 161.78 ± 5.79^3^ | | 156.70 ± 4.79^2,4^ | | 161.55 ± 5.96^3^ | | | **<0.001** | | **<0.001** |
| BMI (kg/m^2^) | 25.96 ± 0.86^2,4^ | | 31.76 ± 4.13^1,3^ | | 26.72 ± 1.26^2,4^ | | 32.11 ± 4.12^1,3^ | | | **<0.001** | | **<0.001** |
| SMM (kg) | 22.08 ± 2.19^2,4^ | | 25.75 ± 3.14^1,3^ | | 22.48±1.95^2,4^ | | 26.26 ±3.52^1,3^ | | | **<0.001** | | **0.002** |
| FFM (kg) | 40.81 ± 3.66^2,4^ | | 46.79 ± 5.09^1,3^ | | 41.44 ± 3.39^2,4^ | | 47.73 ± 5.93^1,3^ | | | **<0.001** | | **<0.001** |
| WHR | 0.86 ± 0.18 | | 1.48 ± 6.98 | | 0.86 ± 0.02 | | 0.94 ± 0.04 | | | 0.68 | | **<0.001** |
| WC (cm) | 84.66 ± 2.16^2,3,4^ | | 101.74 ± 8.99^1,3^ | | 85.02 ± 1.85^2,4^ | | 101.60 ± 8.97^1,3^ | | | **<0.001** | | **<0.001** |
| **Qualitative variables** | | | | | | | | | | | | |
| **Economic status** | | | | | | | | | | | | |
| Poor | | 2(5.0%)^b^ | | 13(32.5%) | 2(5.0%) | 20(57.5%) | | 0.18^***^ | | | 0.65 | |
| Moderate | | 7(7.8%) | | 33(41.9%) | 6(6.6%) | 47(43.7%) | |  |  |  |  |  |
| Good | | 5(5.8%) | | 27(41.3%) | 6(7.1%) | 38(45.8%) | |  |  |  |  |  |
| Excellent | | 1(5.0%) | | 15(75.1%) | 0 (0%) | 4(19.9%) | |  |  |  |  |  |
| **Education status** | | | | | | | | | | | | |
| Illiterate | | 0(0.0%) | | 1(25.0%) | 1(25.0%) | 4(50.0%) | | 0.31 | | | 0.88 | |
| Diploma | | 1(2.0%) | | 17(36.7%) | 2(4.1%) | 31(57.2%) | |  |  |  |  |  |
| University educated | | 14(7.0%) | | 70(43.3%) | 11(6.1%) | 74(43.6%) | |  |  |  |  |  |
| **Marital status** | | | | | | | | | | | | |
| Single | | 5(7.3%) | | 36(52.3%) | 3(3.7%) | 29(36.7%) | | **0.05** | | | 0.47 | |
| Married | | 10(5.9%) | | 52(38.5%) | 11(7.0%) | 80(48.6%) | |  |  |  |  |  |
| **familial obesity history** | | | | | | | | | | | | |
| Yes | | 7 (5.2%) | | 56 (39.7%) | 9 (6.7%) | 85 (48.4%) | | 0.17 | | | 0.92 | |
| No | | 8 (9.3%) | | 32 (47.2%) | 5 (5.5%) | 24 (38%) | |  |  |  |  |  |
| *NWNT: normal waist normal triglyceride, EWNT: enlarged waist normal triglyceride, NWET: normal waist enlarged triglyceride, EWET: enlarged waist enlarged triglyceride,  ^a^Mean±SD; ^b^ N(%), PA: physical activity,* *HOMA-IR: Homeostatic Model Assessment for Insulin Resistance TC: total cholesterol, HDL-C: high density lipoprotein cholesterol, LDL-C: low density lipoprotein cholesterol, TG: triglyceride, SGOT: serum glutamic-oxaloacetic transaminase, SGPT: Serum glutamic-pyruvic transaminase, BMI: body mass index, SMM: skeletal muscle mass, FFM: fat free mass, WHR: waist to hip ratio, WC: waist circumference*  **P value resulted from ANOVA analysis*  *** P value reported after adjusting age, energy intake, BMI and physical activity with ANCOVA analysis*  **** P value resulted from chi-squared test analysis*  *.* | | | | | | | | | | | | |
