# Supplementary material for: Dietary inflammatory index (DII) may be associated with hypertriglyceridemia waist circumference phenotype in overweight and obese Iranian women: a cross sectional study
Source: BMC Res Notes. 2021 Aug 16;14:312. doi: 10.1186/s13104-021-05712-7 (PMC8365886; doi:10.1186/s13104-021-05712-7)
Supplement: Supplementary file 3 — Additional file 3: Table S3. Crude model and adjusted model for Relationship between hyper triglyceridemic waist circumference phenotype and DII. [file 13104_2021_5712_MOESM3_ESM.docx]

**Additional Material**

Additional file 3: Table S3: Crude model and adjusted model for Relationship between hyper triglyceridemic waist circumference phenotype and DII.

| **Table S3: Crude model and adjusted model for Relationship between hyper triglyceridemic waist circumference phenotype and DII** | | | |
| --- | --- | --- | --- |
|  | **OR (****95% CI)** | **β±SE** | **P-trend** |
| **Crude model** | | | |
| EWNT | 1.80(0.91 to1.01) | 0.58±0.34 | 0.08 |
| NWET | 1.74(0.78 to 2.09) | 0.55±0.4 | 0.17 |
| EWET | 2.07(0.42 to 0.69) | 0.73±0.33 | **0.02** |
| **Adjusted model ^a^** | | | |
| EWNT | 2.85(0.77 to 0.86) | 1.04±0.52 | **0.04** |
| NWET | 5.85(0.56 to 0.72) | 1.76±0.85 | **0.03** |
| EWET | 3.13(0.86 to 0.98) | 1.14±0.6 | **0.05** |
| *DII: dietary inflammatory index, OR: odds ratio, CI: confidence interval, SE: standard error, EWNT: enlarged waist normal triglyceride, NWET: normal waist enlarged triglyceride, EWET: enlarged waist enlarged*  *Normal waist normal triglyceride (NWNT) Consider as reference*  *^a^ adjusted for energy intake, age, plasma insulin levels, marriage status, educational status, economic status and familial obesity history* | | | |
